# Supplementary material for: Identification of photoperiod-induced specific miRNAs in the adrenal glands of Sunite sheep (Ovis aries)
Source: Front Vet Sci. 2022 Jul 22;9:888207. doi: 10.3389/fvets.2022.888207 (PMC9354845; doi:10.3389/fvets.2022.888207)
Supplement: Supplementary file 1 [file Data_Sheet_1.ZIP › Supplementary file/miRDeep2 file.zip!/miRDeep2 file/Know_Structure/LP42A2/oar-mir-221.pdf]

miRBase precursor	: oar-mir-221
Total read count	: 3105
oar-miR-221 read count	: 2847
remaining reads	: 258

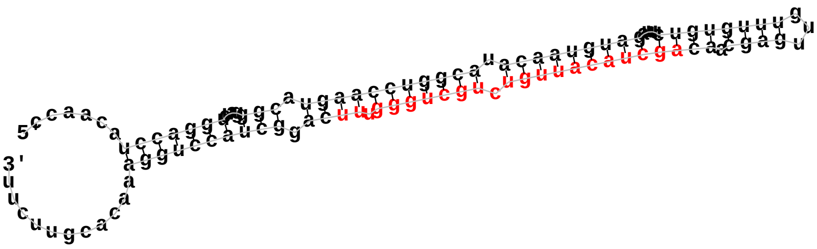

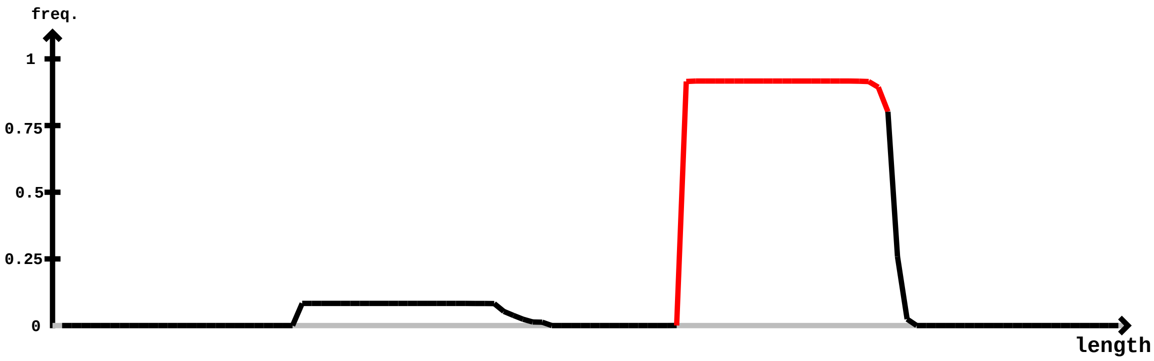

oar-miR-221

```
5'- ccaacauccaggucuagggcaugaaccuggcauacaauguagauuucuguguuuguugagcaacagcuacauugucugcuggguuucaggcuaccuggaaacacguucuu   -3'    exp
    ......((((((....(((.((((((((((((.(((((((((....((((((((...)))).))))))))))))).))))))).)))).))))))))))...........    reads      mm         sample
    ........................accuggcauacaauguag....................................................................    1          0          seq
    ........................accuggcauacaauguagau..................................................................    1          0          seq
    ........................accuggcauacaauguagauu.................................................................    91         0          seq
    ........................accuggcauacaauguagauuu................................................................    47         0          seq
    ........................accuggcauacaauguagauuuc...............................................................    44         0          seq
    ........................accuggcauacaauguagauuucu..............................................................    32         0          seq
    ........................accuggcauacaauguagauuucug.............................................................    3          0          seq
    ........................accuggcauacaauguagauuucugu............................................................    39         0          seq
    ...............................................................cagcuacauugucugcuggguuu........................    1          0          seq
    ................................................................agcuacauugucugcugg............................    2          0          seq
    ................................................................agcuacauugucugcuggg...........................    4          0          seq
    ................................................................agcuacauugucugcugggu..........................    66         0          seq
    ................................................................agcuacauugucugcuggguu.........................    284        0          seq
    ................................................................agcuacauugucugcuggguuu........................    1683       0          seq
    ................................................................agcuacauugucugcuggguuuc.......................    727        0          seq
    ................................................................agcuacauugucugcuggguuuca......................    75         0          seq
    .................................................................gcuacauugucugcuggguu.........................    1          0          seq
    .................................................................gcuacauugucugcuggguuu........................    4          0          seq
```
